# Retrospective analysis of the impact of human papillomavirus infection in the male genital tract on sperm: from a single center

**DOI:** 10.3389/fcimb.2025.1620953

**Published:** 2025-08-20

**Authors:** Huanxin Sun, Xiao Tang, Lingling Tong, Qianqi Liu, Wei Kuang, Wei Wang, Su Zhang, Cheng Wang

**Affiliations:** ^1^ Department of Pathology, West China Second University Hospital of Sichuan University, Chengdu, Sichuan, China; ^2^ Key Laboratory of Birth Defects and Related Diseases of Women and Children (Sichuan University), Chengdu, Sichuan, China; ^3^ Institute of Urology, Key Laboratory of Gansu Province for Urological Diseases, Lanzhou University Second Hospital, Gansu Nephro-Urological Clinical Center, Lanzhou, China

**Keywords:** human papillomavirus, male genital tract, prevalence, semen parameters, fertility

## Abstract

**Objective:**

This study primarily aimed to investigate human papillomavirus (HPV) infection in males and to evaluate its effect on semen parameters, fertility and partner HPV infection status.

**Methods:**

A total of 624 men who visited the West China Second Hospital of Sichuan University between October 1, 2019, and September 30, 2023, were included. HPV DNA was detected in exfoliated cells from the male genitalia using polymerase chain reaction (PCR) and reverse membrane hybridization to analyze the relationship between HPV infection and semen parameters. Furthermore, we retrospectively reviewed medical records of the participants and their partners to collect data on HPV infection and fertility outcomes.

**Results:**

The overall prevalence of HPV infection was 43.8% (273/624), with single-genotype infections accounting for a significantly higher proportion (59.3%, 162/273) than multiple-genotype infections (40.7%, 111/273). The five most prevalent HPV genotypes were HPV52, HPV16, HPV51, HPV58, and HPV42. High-risk (HR) genotypes accounted for most infections (79.5%, 217/273). Among 377 men who underwent semen analysis, HPV-positive individuals exhibited significantly reduced sperm motility and normal morphology compared to HPV-negative individuals (p<0.001). Furthermore, HPV infection was associated with increased sperm DNA fragmentation (p=0.007). Males co-infected with Ureaplasma urealyticum and Chlamydia trachomatis showed significantly lower total sperm counts (×10^6^) (p=0.025) and DFI values (p=0.038) than those without co-infection. Partner data were available for 416 of the 624 men. In these couples, female HPV infection was significantly associated with male HPV status (p=0.038), particularly for HR-HPV (p=0.049). Male HPV-negative status was associated with a higher rate of normal fertility (p<0.001).

**Conclusion:**

Our findings indicate that male genital HPV infection is common and may adversely affect semen quality, fertility, and increase the risk of HPV transmission to sexual partners.

## 1 Introduction

Human papillomavirus (HPV) is one of the most common sexually transmitted infections. Among the more than 170 identified HPV subtypes, over 40 are associated with genital infections. These subtypes are either classified as high-risk (HR-HPV) or non-high risk (non-HR-HPV) based on their oncogenic potential (Graham, 2017; Galeshi et al., 2022). Non-HR-HPV typically causes benign lesions, such as genital and cutaneous warts (Foresta et al., 2015). In contrast, HR-HPV is associated with malignant lesions, including cervical, anal, and oropharyngeal cancers (Soheili et al., 2021). It is estimated that approximately 570,000 women and 60,000 men are infected each year worldwide, with a lifetime probability of infection being 80% for women and 90% for men (Hirth, 2019; Szymonowicz and Chen, 2020). While significant progress has been made in HPV research among women, data on men’s HPV infections remain limited (Lieblong et al., 2019; Tang et al., 2023).

Although HPV-related disease research has historically focused on women, interest in male HPV infection has recently increased. In recent years, several reviews have summarized the related topics of HPV infection in male sperm and sperm quality as well as male infertility (Cao et al., 2020; Garolla et al., 2024). At present, most studies suggest that HPV infection in semen could affect sperm quality and male fertility, but there are still some studies that suggest that HPV infection in semen has no relation to semen quality (Golob et al., 2014; Lyu et al., 2017). This difference might be related to the limitations of various research investigations (including differences in the study population, semen microenvironment, co-infection with other specific pathogenic microorganisms, or other factors).

Ejaculation dysfunction is a relatively serious problem in male infertility, accounting for 14% to 18% of infertile men (Álvarez et al., 2024). For certain men who struggle to obtain semen smoothly, sampling from the genital tract becomes especially crucial. Despite the potential for contamination, the impact of HPV on the genital tract has been investigated much less in men than in women. This might be due to the fact that men do not have specific symptoms and the limitations of methods for assessing HPV infection in the male genital tract. Therefore, there is relatively little data on the prevalence of HPV in the male genital tract. Literature reports indicate that the detection rate of HPV in semen and the genital tract is consistent (Olivera et al., 2024). However, there are few literatures that statistically analyze the series of impacts caused by HPV infection in the male genital at present.

The series of effects of HPV infection in male genital tract remain an area of active investigation. This study aimed to evaluate the prevalence of genital HPV infection in men, assess the distribution of HPV subtypes, and investigate the association between HPV infection including co-infection with other pathogens and semen parameters. Meanwhile, analyze the consistency of HPV infection in the male genital tract and that in the spouse. These findings may help elucidate the potential adverse effects of HPV on male genital health.

## 2 Materials and methods

### 2.1 Study population

**Figure 1:**
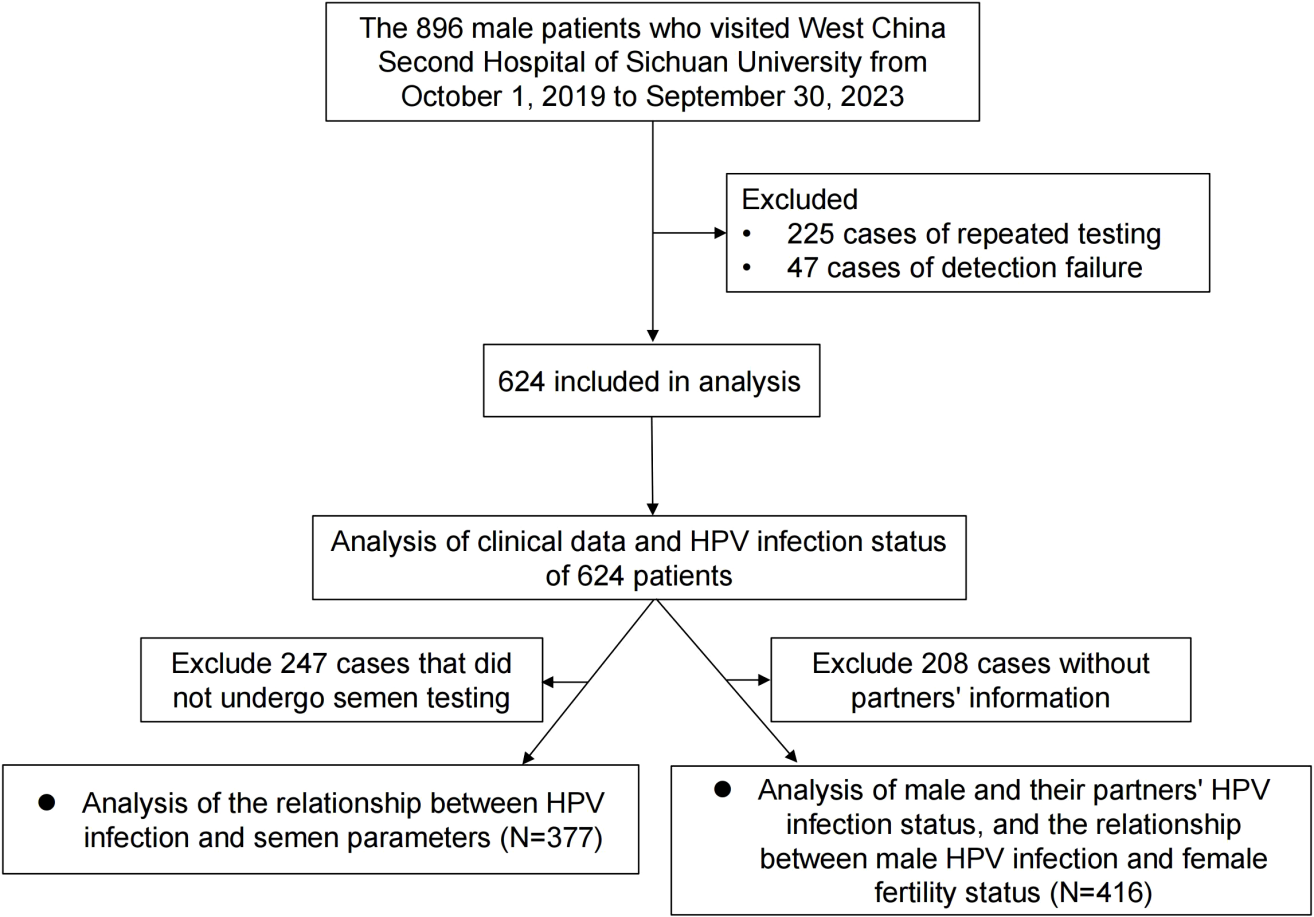
Flow diagram outlining the study’s selection process and overall workflow.

In this retrospective study, we enrolled 896 male patients who visited the West China Second Hospital of Sichuan University between October 1, 2019, and September 30, 2023. Of these, a total of 225 patients were excluded due to duplicate HPV testing, and 47 were excluded due to unsuccessful HPV tests. Ultimately, 624 patients were included in the final analysis. We recorded each participant’s age, ethnicity, educational level, marital status, sampling site, circumcision history, and reason for testing. Among the 624 patients, we also collected information on the female partners of 416 men who underwent HPV testing within a 3-month interval of their partner’s test. Female partners with confirmed tubal, uterine, cervical, or ovarian abnormalities were excluded. Additionally, 377 of the 624 male patients underwent semen analysis. The evaluated semen parameters included total sperm count (×10^6^), sperm concentration (/mL), progressive motility (%), normal morphology (%), DNA fragmentation index (DFI), and the presence of Ureaplasma urealyticum (UU) and Chlamydia Trachomatis (CT). Males with known genetic diseases or severe inflammatory conditions were excluded from the analysis on semen parameters to avoid confounding effects. The inclusion criteria are outlined in the flow diagram presented in **Figure 1**. This study was approved by the Ethics Committee of the West China Second Hospital of Sichuan University (Approval No. 2024198).

### 2.2 HPV detection and genotyping

All specimens were collected by specialized doctors, and the collection sites were mainly located at the male urethral orifice, glans penis, coronal sulcus. Cells were stored in standard preservative media provided by the manufacturer of Yaneng biotechnology (Shenzhen, China). DNA extraction was performed with a NX-48 swab DNA kit (Seoul, Korea) according to the manufacturer’s instructions. The DNA was subjected to GP5þ/6þ polymerase chain reaction (PCR), subsequent microsphere bead-based genotyping. Fourteen HR-HPV genotypes (16, 18, 31, 33, 35, 39, 45, 51, 52, 56, 58, 59, 66, 68) and nine non-HR-HPV genotypes (HPV 6, 11, 42, 43, 53, 73, 81, 82, 83) were classified using in reverse membrane dot blot hybridization (Zhang et al., 2018). Finally, positive results appeared as a blue dot observable by the naked eye on the strip. Negative and positive controls are set up for each batch to ensure accurate and reliable results.

### 2.3 Sperm parameters

Patients should restrain from sexual activity for 2–7 days before testing. Sperm should be collected through masturbation, and the collected semen should be placed in a 37°C water bath. After complete liquefaction, semen routine and other testing items should be performed. professional doctors and related technical personnel jointly interpret semen reports. The WHO Manual for the Laboratory Examination and Processing of Human Semen (6^th^ edition) was used for the semen analysis (Björndahl et al., 2022). They were centrifuged at 4°C (2000×g, 20 min) to separate spermatozoa from seminal plasma. The sampling interval between penile scraping specimens and semen samples for all patients was less than one week. Defined progressive motility<32% as reduced sperm viability, also known as asthenospermia. Teratospermia is diagnosed when the proportion of sperm with normal morphology falls below <4%. And oligospermia is defined as sperm count of <15×10^6^ in one ejaculation.

### 2.4 Statistical analysis

Data analysis was performed using SPSS software (version 22.0, Chicago, USA) or GraphPad Prism software (version 9.0, San Diego, USA). Categorical variables were presented as frequencies and percentages. Normally distributed variables were expressed as mean ± standard deviation (s.d.). Independent samples t-test and Pearson Chi-square analysis were employed for continuous and categorical variable differences, respectively. Analysis of variance was used for comparisons between multiple groups, with significance set at P<0.05.

## 3 Results

### 3.1 Prevalence and genotype distribution of HPV

**Figure 2:**
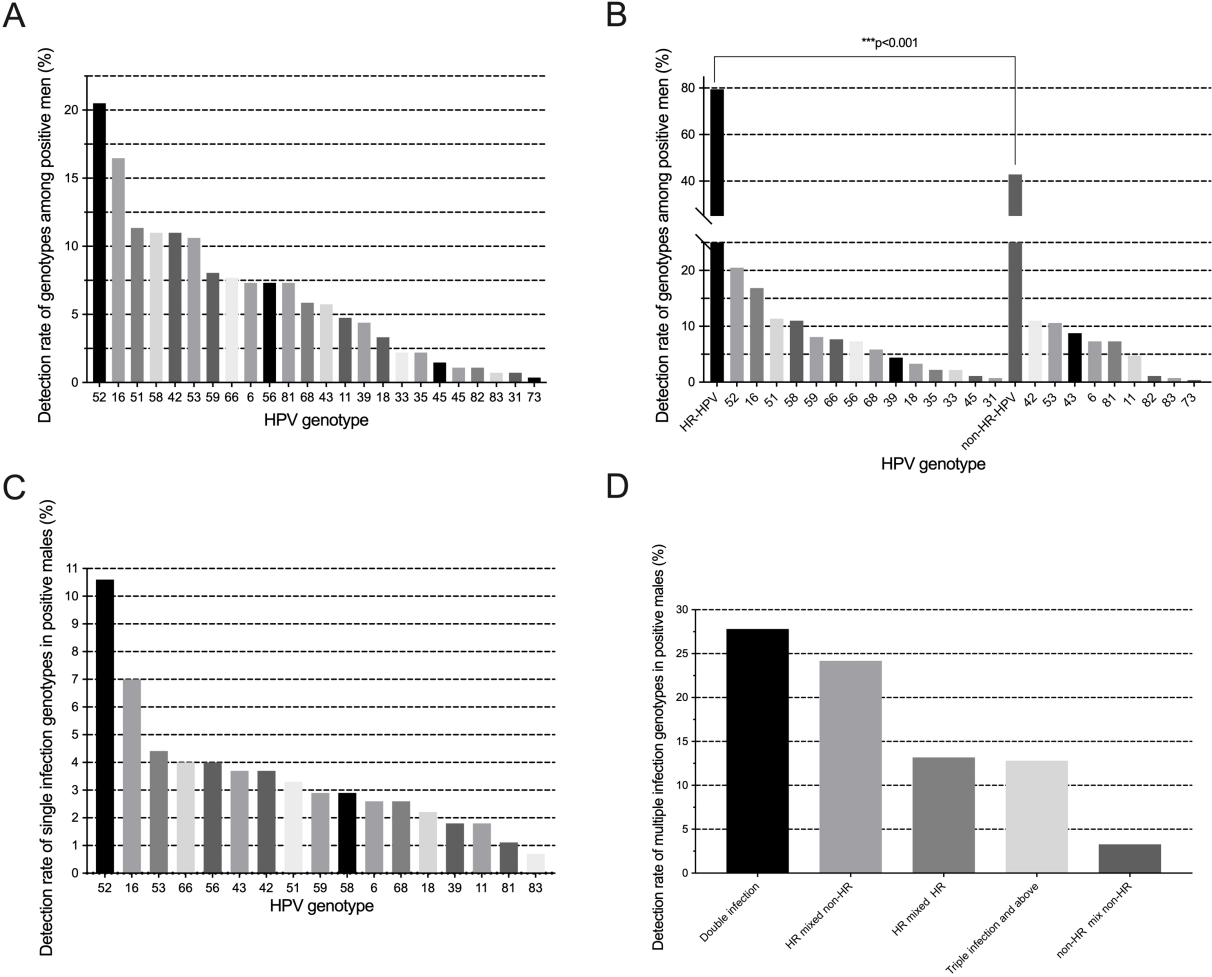
**(A)** Frequency distribution of HPV genotypes detected; **(B)** Prevalence and comparisons of HR/non-HR genotypes; **(C)** Distribution of single-genotype infections; **(D)** Patterns of multiple-genotype HPV infections.

As shown in **Figure 2A**, the 23 genotypes were all detected. The overall rate of HPV infection was 43.8% (273/624). The most prevalent genotypes were HPV52 (20.5%, 56/273), HPV16 (16.5%, 45/273), HPV51 (11.4%, 31/273), HPV58 (11.0%, 30/273), and HPV42 (11.0%, 30/273). HR-HPV genotypes infection is the most common infection (79.5%, 217/273). There was a significant difference between the prevalence of HR and non-HR (**Figure 2B**). More than half of the males were detected with a single HPV genotype infection (59.3% 162/273). The prevalence rate of single HPV infection in males is shown in **Figure 2C**. Among multiple HPV genotype infections, 68.5% (76/111) of men were detected to have double infection. Among multiple infections, HR-HPV mixed non-HR-HPV infection is the most common infection pattern (**Figure 2D**).

### 3.2 Baseline characteristics of study participants

A total of 624 men aged 18–59 years were included in this study, and the mean age was 34.2 ± 7.6 years. **Table 1** summarized the basic clinical information of men, according to Chi-square test, there was no statistically significant difference in age, ethnic, education level, marriage status, sampling site, previous circumcision, and reason for testing with HPV infection. However, the highest prevalence of HPV infection was observed in men aged 30–39 years, accounting for nearly half of all infections (47.6%, 130/273). Additionally, a substantial proportion of HPV-positive men (71.8%, 196/273) had an educational level of a bachelor’s degree or higher.

**Table 1:** Baseline characteristics of the study participants (n=624).

Variables	Negative	Positive	Overall	p-value
	(N=351)	(N=273)	(N=624)	
Age				0.298
18-29	117 (33.3%)	83 (30.4%)	200 (32.1%)	
30-39	165 (47.00%)	130 (47.6%)	295 (47.3%)	
40-49	51 (14.5%)	36 (13.2%)	87 (13.9%)	
50-59	18 (5.1%)	24 (8.8%)	42 (6.7%)	
Ethnic				
Han	322 (91.7%)	250 (91.6%)	572 (91.7%)	0.792
Hui	5 (1.4%)	5 (1.8%)	10 (1.6%)	
Tibetan	13 (3.7%)	8 (2.9%)	21 (3.4%)	
Uygur	3 (0.9%)	5 (1.8%)	8 (1.3%)	
Yi	8 (2.3%)	5 (1.8%)	13 (2.1%)	
Education level				
Bachelor degree or above	231 (65.8%)	196 (71.8%)	427 (68.4%)	0.166
Junior school	14 (4.0%)	13 (4.8%)	27 (4.3%)	
Senior school	106 (30.2%)	64 (23.4%)	170 (27.2%)	
Marriage status				0.142
Married/Living with partner	212 (60.4%)	148 (54.2%)	360 (57.7%)	
Single/Never Married	139 (39.6%)	125 (45.8%)	264 (42.3%)	
Reason for Test				0.142
Fertility	109 (17.5%)	59 (9.5%)	168 (26.9%)	
Physical	200 (32.1%)	174 (30%)	374 (59.9%)	
Genital warts	42 (6.7%)	40 (6.4%)	82 (13.2%)	
Sampling sites				0.485
Urethral orifice	144 (23.1%)	109 (17.5%)	253 (40.6%)	
Glans	121 (19.4%)	95 (15.2%)	216 (34.6%)	
Coronal sulcus	86 (13.8%)	69 (11.1%)	155 (24.8%)	
Previous circumcision				0.334
Yes	272 (77.5%)	166 (60.8%)	438 (70.2%)	
No	79 (22.5%)	107 (39.2%)	186 (29.8%)	

### 3.3 Association of male HPV infection with female partner HPV status

A total of 416 female partners were included in the analysis. Among HPV-positive males, the prevalence of any HPV genotype in their partners was 65.9%, with 79.9% testing positive for HR-HPV genotypes and 36.0% for non-HR-HPV genotypes. The most frequently detected genotypes in women were HPV16 (31.7%, 57/180), HPV52 (31.1%, 56/180), and HPV58 (22.8%, 41/180). Among non-HR-HPV genotypes, HPV53 had the highest prevalence (27.8%, 50/180). Partner HPV infection was significantly associated with male HPV status (p=0.038). Female partners of HPV-positive men had a higher prevalence of HR-HPV infection compared to partners of HPV-negative men (79.9% vs. 61.8%, p=0.049; **Table 2**).

**Table 2:** The influence of male HPV status on the distribution of HPV genotypes in females.

Female HPV genotypes	Status of HPV infection in males		p-value
	HPV positive (n=189)	HPV negative (n=227)	
Any HPV(n=363)	95.2% (180/189)	80.6% (183/227)	0.038
HR-HPV (n=291)	79.9% (151/189)	61.8% (140/227)	0.049
HPV16/18(n=65)	17.5% (33/189)	14.1% (32/227)	0.155
HPV51/52/58(n=142)	45.5% (86/189)	24.7% (56/227)	0.179
Non-HR-HPV(n=141)	36.0% (68/189)	32.2% (73/227)	0.618
HPV53(n=48)	13.8% (26/189)	9.7% (22/227)	0.273
HPV6/11(n=39)	10.6% (20/189)	8.4% (19/227)	0.650
HPV42/43/81(n=75)	21.2% (40/189)	15.4% (35/227)	0.526

Any HPV: Positive for any type of HPV; HR-HPV: Positive for any genotype containing 16, 18, 31, 33, 35, 39, 45, 51, 52, 56, 58, 59, 66, 68 classified as HR-HPV; HPV16/18: Positive for any genotype containing 16, 18; HPV51/52/58: Positive for any genotype containing 51, 52, 58; Non-HR-HPV: Positive for any genotype containing 6, 11, 42, 43, 53, 81, 73,82, 83 classified as non-HR-HPV**;** HPV6/11: Positive for any genotype containing 6, 11; HPV42/43/81: Positive for any genotype containing 42, 43, 81.

**Figure 3:**
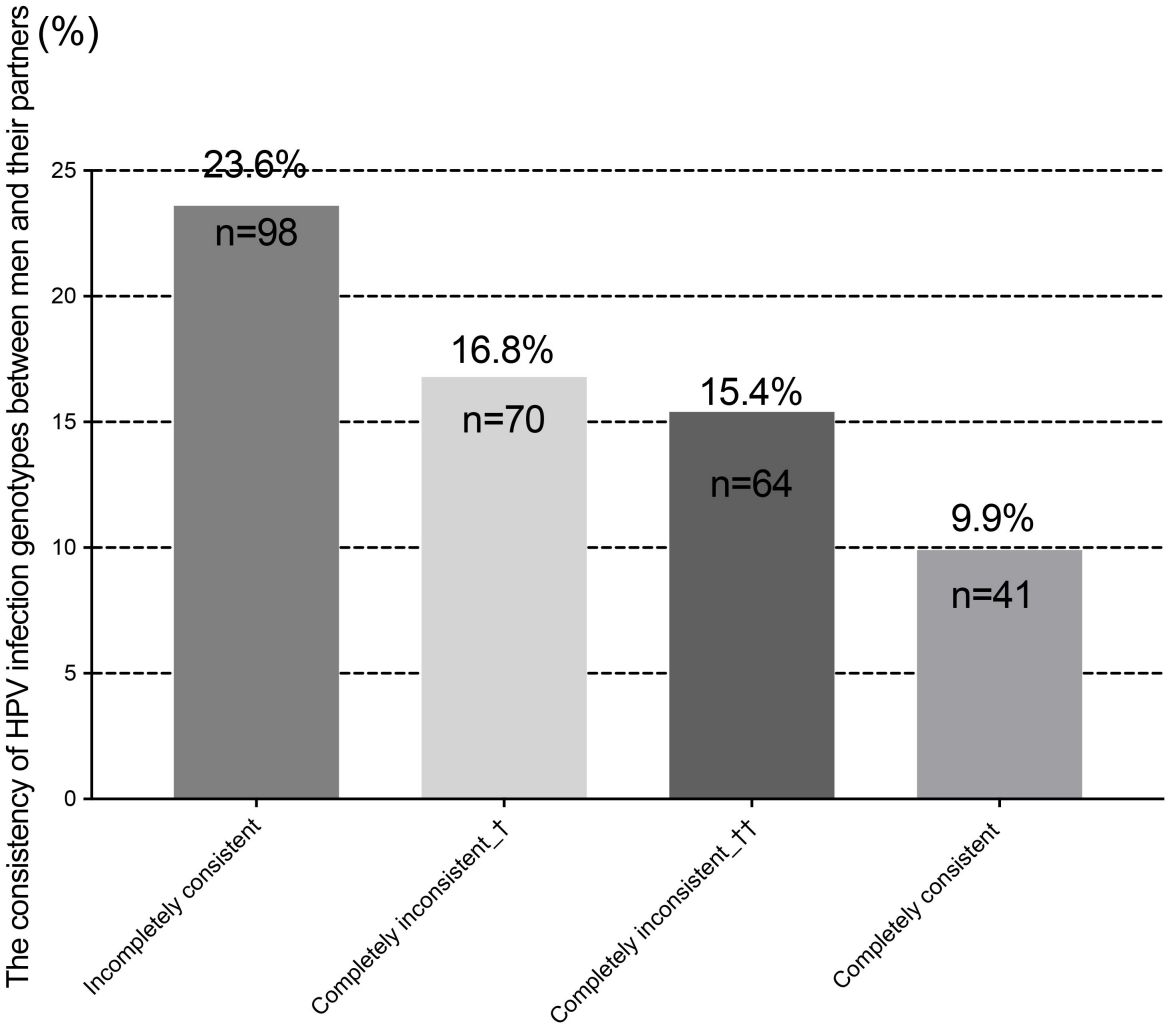
Concordance between male and partner HPV genotypes. Completely inconsistent +: Genotypes detected in male HPV infection and partner HPV infection differ. Completely inconsistent ++: Male is HPV-positive; female is HPV-negative.

We further analyzed the concordance of HPV genotypes between male participants and their partners. Of the 416 couples, 41 (9.9%) had concordant genotypes, 98 (23.6%) had partially concordant genotypes, and 134 (32.2%) were completely discordant in HPV infection status. Notably, 81 men (19.5%) were infected with genotypes completely different from those found in their partners. In 53 couples (12.7%), the male was HPV-positive while the female was HPV-negative (**Figure 3**). These findings indicate that male genital HPV positivity increases the risk of HPV infection in their female partners, particularly for HR-HPV genotypes.

### 3.4 Effect of HPV infection on semen parameters

Among the 377 men who underwent semen analysis, we compared parameters between HPV-positive and HPV-negative individuals (**Table 3**). HPV-positive men demonstrated significantly lower sperm motility and a reduced proportion of morphologically normal sperm (p<0.001). Additionally, the sperm DFI was significantly higher in HPV-positive individuals (p=0.007). However, no significant differences were observed in total sperm count (×10^6^) or sperm concentration (/mL).

**Table 3:** Comparison of semen parameters between HPV-positive and negative patients (n=377).

Sperm parameter	HPV-positive	HPV-negative	p-value
Total sperm number (×10^6^)	235.2 ± 13.7	271.9 ± 14.3	0.078
Sperm Concentration/mL	84.5 ± 4.5	92.0 ± 3.2	0.165
PR (%)	40.8 ± 1.3	49.0 ± 1.0	<0.001
Normal forms (%)	5.8 ± 0.2	7.5 ± 0.2	<0.001
DFI	15.6 ± 0.4	12.1 ± 0.1	0.007

PR, progressive motility(A+B); DFI, DNA fragmentation index.

We further examined the relationship between HPV infection and fertility status. A higher proportion of HPV-negative men had normal fertility compared to HPV-positive men (39.4% vs 26.2%, p<0.001). However, there were no significant associations between HPV status and either miscarriage rates (9.6% vs 7.7%) or infertility rates (9.9% vs 7.2%) (**Supplementary Table 1**). Neither HR-HPV nor non-HR-HPV subtypes were associated with significant changes in semen parameters. Additionally, no significant differences in sperm parameters were observed between men with single-genotype versus multiple-genotype HPV infections (p > 0.050) (**Supplementary Tables 2, 3**).

Males with HPV combined with UU and CT positivity had significantly lower total sperm counts (×10^6^) (p=0.025) and DFI values (p=0.038) compared to those with UU/CT positivity alone. However, no statistically significant differences were observed between the HPV-only and UU/CT-only groups across these parameters. Notably, when UU infection was present, concurrent HPV infection significantly reduced total sperm count (p=0.011), as shown in **Table 4**.

**Table 4:** Sperm parameter in men with HPV infection and pathogenic coinfections (n=377).

Sperm parameter	HPV-positive alone	Pathogen coinfections alone	p-value	HPV positive and pathogen coinfections	HPV negative and pathogen coinfections	p-value	HPV-positive and UU positive	HPV-negative and UU positive	p-value
Total sperm number (×106)	240.5 ± 12.7	267.8 ± 14.4	0.092	215.6 ± 11.8	267.8 ± 14.4	0.025	208 ± 10.9	261.9 ± 12.9	0.011
Sperm Concentration/mL	85.6 ± 4.6	90.4 ± 5.8	0.185	83.9 ± 4.4	90.4 ± 5.8	0.155	82.8 ± 4.4	91.9 ± 6.0	0.096
PR (%)	38.9 ± 3.6	41.7 ± 4.2	0.269	35.8 ± 3.1	41.7 ± 4.2	0.142	36.2 ± 3.4	41.1 ± 4.2	0.106
Normal forms (%)	5.5 ± 1.0	5.7 ± 1.0	0.411	4.6 ± 0.8	5.7 ± 1.0	0.226	4.2 ± 0.7	6.0 ± 1.1	0.157
DFI	12.5 ± 1.2	12.6 ± 1.2	0.488	15.2 ± 1.6	12.6 ± 1.2	0.038	14.3 ± 1.4	13.8 ± 1.3	0.264

PR, progressive motility(A+B); DFI, DNA fragmentation index; Pathogen coinfections alone: Only positive for either or both of the UU CTs; HPV positive and pathogen coinfections: HPV positivity combined with either UU or CT positivity or both HPV UU CT positivity; HPV negative and pathogen coinfections: Only positive for either or both of the UU and CTs.

## 4 Discussion

HPV infection is a major public health concern, particularly among women, for whom extensive evidence exists regarding its epidemiology and associated health impacts. However, our understanding of HPV epidemiology in men remains limited (Lieblong et al., 2019). In recent decades, there has been increasing interest in male HPV infections and their potential effects on sperm quality and fertility (Cao et al., 2020; Rivero et al., 2023; Garolla et al., 2024). In this retrospective study involving 624 males from Sichuan, China, we evaluated the prevalence and genotype distribution of genital HPV infections, concordance of HPV genotypes between couples, and associations between HPV infection status and semen parameters.

As one of the most common sexually transmitted pathogens, HPV prevalence in men worldwide ranges from 1.3% to 72.9% (Dunne et al., 2006). In our cohort, the overall prevalence was 43.8% (273/624), slightly higher than the 31% male infection rate reported in a 2023 study across 35 countries (Bruni et al., 2023). Single-genotype HPV infection was the most common pattern in our study population, accounting for 59.3% (162/273), compared to dual (27.8%) and multiple (≥ three genotypes, 13.6%) infections. This result aligns with findings from other regions (Abbasi et al., 2024; Liu et al., 2024), suggesting that HPV infection in men in Sichuan predominantly presents as a single-type infection. A total of 23 HPV genotypes were identified, with HPV52 (20.50%) and HPV16 (16.50%) being the most common HR-HPV types—findings largely consistent with previous reports (Huang et al., 2024; Wen et al., 2025). HPV42 was the most prevalent non-HR-HPV genotype in our cohort, whereas other studies more commonly report HPV6 or HPV11 as dominant (Szymonowicz and Chen, 2020). Non-HR-HPV genotypes may cause genital or cutaneous warts. In particular, HPV42 has been associated with low-grade cervical intraepithelial lesions in women. (28–30). The fact suggests that the different genotypes of prevalence vary from one geographic area to another. The HPV genotypes included in the Gardasil 9 vaccine (6, 11, 16, 18, 31, 33, 45, 52, and 58) represent 65.2% of those detected in our cohort. We findings indicate that male genital HPV positivity increases the risk of HPV infection in their female partners, particularly for HR-HPV genotypes.

Although the types of infections between partners may be similar. The Benevolent team’s study has confirmed that male HPV positivity is more common in HPV-positive female sexual partners. Males may be an important source of HPV transmission between sexual partners (Benevolo et al., 2008). Thus, the vaccination of males against HPV might be a prevention option. Our study offers preliminary insights into HPV prevalence and genotype distribution among reproductive-age men in Sichuan.

HPV may persist in the genital tract, including semen, male external genitalia, perianal skin, urethra, vas deferens, epididymis, and testes, and continue to infect the host (Dunne et al., 2006). Reports assessed the relationship between male fertility and HPV infection in semen. It showed that HPV infection in semen impairs male fertility to a certain extent and that HPV in semen alters some parameters of spermatozoa, especially sperm motility (Xiong et al., 2018; Santos et al., 2023). Our research found that genital tract HPV-positive men demonstrated significantly lower sperm motility and a reduced proportion of morphologically normal sperm. HPV infection could affect every cell in the body, including sperm count, motility, genome integrity, morphology, and concentration (Schillaci et al., 2013). Although the specific mechanism of HPV infection in semen is currently unclear, and the role of infected cells in virus transmission is also vague, but it can be confirmed that HPV virus can bind to the equatorial end of sperm head through the interaction between L1 viral capsid protein and proteoglycan Syndecan-1, thereby affecting sperm motility (Foresta et al., 2011; Schillaci et al., 2013). However, some studies have reported no association between HPV infection in semen and impaired sperm function (Bezold et al., 2007; Foresta et al., 2015; Luttmer et al., 2016). Such discrepancies in findings may stem from differences in study populations, semen microenvironments, or other factors.

Roosmarijn et al. found that 85% of men had consistent HPV infections in their genital and semen specimens. This was determined by taking scrapings from the penis and three semen samples, with each sample taken one week apart (Luttmer et al., 2015). The presence of HPV in semen was associated with the presence of HPV in the penile scrape also on a genotype-specific level. The researchers studied 213 cases of HPV infection in men, both on the penis and in the sperm. They found that men who tested positive for HPV in penile scrapings were more likely to test positive for HPV in semen than those who tested negative. At the same time, it was found that the semi-quantitative HPV viral load in penile scratches was statistically positively correlated with the semi-quantitative viral load in semen (Luttmer et al., 2015). We suspect that the process of sperm infection with HPV might be a downward infection through the male urogenital tract and sexual behavior. Our research indicates that HPV infection in the genital tract may have a negative impact on semen, there is no statistical difference between HR-HPV and non-HR-HPV infections and sperm. This is similar to the research results of Carolina et al (Olivera et al., 2024). However, there are contrary studies suggesting that we should focus on the specific types of HPV infection in the male genital tract. Though neither HR-HPV nor non-HR -HPV were associated with significant alterations in routine sperm quality parameters. HR-HPV+ individuals showed significantly higher levels of sperm necrosis and exhibited increased proportions of ROS+ spermatozoa compared to non-HR-HPV+ or control individuals (Olivera et al., 2024). Despite this, the impact of HR or non-high-risk on sperm remains unclear. Some reports suggest that HR-HPV is more likely to have adverse effects on sperm, as sperm DNA fragmentation is more likely to lead to an increase in sperm necrosis (Boeri et al., 2019; Capra et al., 2022).

HPV infection may have adverse effects on sperm and, by extension, negatively impact male fertility. For instance, HPV16 and HPV31 have been shown to impair embryo development, while HPV11, HPV16, HPV18, and HPV31 reduce implantation rates (Zacharis et al., 2018). In contrast, Tangal et al. reported no significant differences in implantation or clinical pregnancy rates between individuals with HPV-infected and uninfected sperm undergoing *in vitro* fertilization (IVF). However, the miscarriage rate in IVF procedures involving HPV-infected spermatozoa was reported to be 33%, compared to 10% in uninfected individuals (Tangal et al., 2019). In our study, although the associations between HPV infection and either infertility or miscarriage were not statistically significant, HPV-positive men still had numerically higher rates of both outcomes than HPV-negative men. Prior studies have also reported reduced pregnancy rates and increased miscarriage rates in both male and female HPV-infected individuals (Garolla et al., 2016). Depuydt et al. found that pregnancy rates following intrauterine insemination declined when sperm DFI exceeded 26%, and that sperm samples containing HPV had significantly higher DFI values than HPV-negative samples (Depuydt et al., 2021). Other studies have also demonstrated that HR-HPV infection is associated with decreased semen viscosity, reduced sperm motility, and increased sperm DNA fragmentation (Damke et al., 2017; Boeri et al., 2019). Our results show that male urogenital infection by either HR-HPV or non-HR-HPV does not impair most sperm parameters. This is consistent with the viewpoint of Canarella et al (Cannarella et al., 2022). This difference might be due to deviations in our analytical methods. Because our high-risk type infection is defined as any infection type that includes any high-risk genotype. Therefore, the high-risk HPV infections we included in the analysis may also include other non-high-risk types. Subsequently, we analyzed single-type HPV infection and multi-type HPV infection in the male genital tract, and the results showed that there was no difference in their sperm parameters. Nonetheless, the overall impact of HPV infection on male fertility and reproductive outcomes remains controversial. However, further studies will be needed to assess the infectiousness of genital HPV and its impact on reproductive health.

Interestingly, in the semen microenvironment, it is worthwhile to pay attention to co-infections with other pathogenic microorganisms, and in this paper, we analyzed the effect of HPV co-infections with the most common pathogenic microorganisms, UU and CT, on semen in men, in order to exclude the potential effect of other concurrent infections. We found that coinfection with HPV significantly lowered total sperm number when the above-mentioned common infections were present. In addition, total sperm number were significantly reduced in men with HPV mixed UU infection. Evidence suggests that semen biota play an important role in determining reproductive health and pregnancy outcomes. However, unlike other studies, we did not find that UU and CT infections were associated with sperm viability. This may stem from the diversity of the semen microenvironment and the *in vivo* balance of microorganisms, and perhaps studies with larger samples are needed to clarify the effect of HPV coinfection on semen.

Our study has several strengths. First, we collected extensive clinical data to maximize the exclusion of confounding factors that may interact with HPV infection. Second, our sampling was focused primarily on the external genitalia, particularly the urethra. Most existing studies have concentrated on detecting HPV in semen and examining its effects on semen parameters and male fertility. However, few have explored the series of impacts of genital HPV infection on men. At present, there is no gold standard for sampling. Differences in sampling techniques across studies may partially explain the variability in reported infection rates. Nonetheless, genital sampling in me should be promoted, as it is quick, painless, and minimally invasive.

This study also had some limitations. First, we did not analyze the correlation between HPV infection of the external genitalia and HPV presence in semen. Prior research has shown that HPV prevalence and viral load can vary depending on the anatomical sampling site (Hernandez et al., 2008). Genital epithelial scraping specimens are more likely to be HPV-positive than semen samples (Luttmer et al., 2015), which may have contributed to a slightly higher positivity rate in our male cohort. Furthermore, all participants were first-time testers, and it is possible that some had recently cleared transient HPV infections, thereby introducing potential bias into our statistical analysis. Finally, for patients whose partners experienced infertility or miscarriage, we could not fully rule out female-related causes, which may have confounded our evaluation of the relationship between HPV infection and fertility outcomes.

## 5 Conclusion

We indicated that HPV genital tract infection in men is a widespread phenomenon, and it is also a serious social problem. Male infection with HPV not only increases the risk of transmission to partners but also affects semen quality and harms fertility. Given its simplicity and speed, genital tract HPV screening could be integrated into routine health assessments for men, providing valuable insights into male reproductive health and contributing to broader HPV surveillance efforts.

## Data Availability

The original contributions presented in the study are included in the article/**Supplementary Material**. Further inquiries can be directed to the corresponding author.
