# Supplementary material for: Retrospective analysis of the impact of human papillomavirus infection in the male genital tract on sperm: from a single center
Source: Front Cell Infect Microbiol. 2025 Aug 20;15:1620953. doi: 10.3389/fcimb.2025.1620953 (PMC12405403; doi:10.3389/fcimb.2025.1620953)
Supplement: Supplementary file 1 [file Table1.docx]

**Supplementary table 1.** The influence of male infection on the fertility status in females (n=416).

| Female fertility | Status of HPV infection in males(n=416) | | p-value |  |
| --- | --- | --- | --- | --- |
|  | HPV positive | HPV negative |  |  |
| Normal | 109(26.2%） | 164(39.4%) | ＜0.001 |  |
| Infertile | 40(9.6%) | 32(7.7%) | 0.155 |  |
| Miscarriage | 41(9.9%) | 30(7.2%) | 0.218 |  |

**Supplementary table 2.** Semen parameters analysis between HR‑HPV and non-HR‑HPV infection(n=377).

| Sperm parameter | HR-HPV | Non-HR-HPV | p-value |
| --- | --- | --- | --- |
| Sperm concentration/mL | 85.6±3.5 | 88.6±3.2 | 0.482 |
| Total sperm number (×10^6^) | 72.4±3.8 | 69.5±3.3 | 0.571 |
| PR (%) | 40.4±1.5 | 39.1±2.4 | 0.641 |
| Normal forms (%) | 5.8±0.2 | 5.9±0.3 | 0.713 |
| DNA fragmentation index (DFI) | 12.7±0.2 | 12.3±0.3 | 0.197 |

PR (%)：progressive motility(A+B); DFI：DNA fragmentation index

**Supplementary table 3.**

| Semen parameters analysis between Single infection and Multiple infections infection(n=377). | | | |
| --- | --- | --- | --- |
| Sperm parameter | Single  infection | Multiple   infections | p-value |
| Sperm concentration/mL | 76.6±5.5 | 74.8±5.2 | 0.581 |
| Total sperm number (×10^6^) | 234.4±12.8 | 233±11.9 | 0.825 |
| PR (%) | 41.4±1.9 | 39.8±2.3 | 0.608 |
| Normal forms (%) | 5.5±0.2 | 5.8±0.3 | 0.712 |
| DNA fragmentation index (DFI) | 12.4±0.3 | 12.9±0.2 | 0.481 |

PR (%)：progressive motility(A+B); DFI：DNA fragmentation index
